# Acupuncture at local and distal points for chronic shoulder pain: study protocol for a randomized controlled trial

**DOI:** 10.1186/1745-6215-15-130

**Published:** 2014-04-17

**Authors:** Qing-Nan Fu, Guang-Xia Shi, Qian-Qian Li, Tian He, Bao-Zhen Liu, San-Feng Sun, Jun Wang, Cheng Tan, Bo-Feng Yang, Cun-Zhi Liu

**Affiliations:** 1 Acupuncture and Moxibustion Department, Beijing Hospital of Traditional Chinese Medicine affiliated to Capital Medical University, 23 Meishuguanhou Street, Dongcheng District, Beijing 100010, China; 2 Shandong University of Traditional Chinese Medicine, Jingshi Street, Lixia District, Jinan 250014, China; 3 Beijing Huairou District Hospital of Chinese Medicine, 1 Houheng Street jia, Huairou District, Beijing 101400, China; 4 Dongzhimen Hospital Affiliated to Beijing University of Chinese Medicine, 5 Haiyuncang, Dongcheng District, Beijing 100010, China

## Abstract

**Background:**

Chronic shoulder pain (CSP) is the third most common type of musculoskeletal pain. It has a major impact on health-related quality of life. In Chinese medicine, CSP is considered one of the conditions most amenable to treatment with acupuncture. The purpose of this study is to evaluate the efficacy of local acupoints in combination with distal acupoints in pain relief and shoulder function improvement in CSP patients.

**Methods/Design:**

This is a multicenter, single blind, factorial randomized controlled clinical trial. A total of 164 participants will be randomly allocated to four different groups: Group A will receive acupuncture at local acupoints in combination with distal acupoint. Group B will receive acupuncture at local acupoints in combination with distal non-acupoint. Group C will receive acupuncture at local non-acupoints in combination with distal acupoint. Group D will receive acupuncture at local non-acupoints in combination with distal non-acupoint. Each group will receive 12 treatments of acupuncture one to three times per week for six weeks in total. The primary outcome is shoulder pain intensity, which is graded using a 100 -mm Visual Analogue Scale. The assessment is at baseline (before treatment initiation), 6 weeks after the first acupuncture, 10 weeks after the first acupuncture and 18 weeks after the first acupuncture.

**Discussion:**

This trial will be helpful in identifying whether acupuncture at local acupoints in combination with distal acupoints may be more effective than needling points separately.

**Trial registration:**

International Standard Randomized Controlled Trial Number Register: ISRCTN61861069 (http://www.controlled-trials.com).

## Background

Chronic shoulder pain (CSP) is the third most common type of musculoskeletal pain. It has a major impact on health-related quality of life [1]. Disorders of the shoulder muscles and tendons (rotator cuff) are thought to be the most common causes of the pain [2]. The true prevalence is unknown, varying in different reports from 4 to 34% [3]. The conventional treatments for shoulder pain are NSAIDs, physiotherapy, cortisone injections and ‘wait and see’ [4,5]. Unfortunately, none of these treatments are clearly proven to be effective for CSP in the long run [4-6]. In Chinese medicine, CSP is considered one of the conditions most amenable to treatment with acupuncture [4]. The purpose of this study is to evaluate whether local acupoints in combination with distal acupoints are more effective than local acupoints or distal acupoints alone in pain relief and shoulder function improvement in CSP patients.

## Methods/Design

### Ethics

The study is in accordance with the Declaration of Helsinki and has been approved by the Research Ethical Committee of Beijing Hospital of Traditional Chinese Medicine Affiliated to Capital Medical University(reference: 201315). This trial was registered with ISRCTN at Current Controlled Trials (ISRCTN61861069). Before randomization all patients will be requested to sign a written informed consent, and they will also be given enough time to decide whether they are willing to participate in the trial or choose other treatment options.

### Population

Patients will be recruited in acupuncture clinics in the Beijing Hospital of Traditional Chinese Medicine Affiliated to Capital Medical University, the Beijing Huairou District Hospital of Traditional Chinese Medicine, and the Dongzhimen Hospital Affiliated to Beijing University of Chinese Medicine, with a target sample size of 164 subjects. The trial will be executed from January 2014 to December 2015.

### Recruitment of participants

Two strategies will be used to recruit participants with CSP. One is to recruit participants in outpatient clinics from the three hospitals mentioned above. The other is to show recruitment posters outside the clinics. The posters will contain brief introductions about the population needed, the free acupuncture treatments offered to eligible participants, and the contact information of the researcher. Screening forms will be completed by interested patients, which will be conducted via a face-to-face interview and assessment with a researcher at one of the hospital sites prior to randomization.

### Inclusion criteria

Patients who meet all of the following conditions will be considered for enrollment. The inclusion criteria are as follows: aged between 25 and 65 years (either sex); present with a primary complaint of shoulder pain with one-sided shoulder pain for at least six weeks and up to two years; a pain score of 50 mm or more on a 100 -mm visual analogue scale (VAS); plain radiography is normal, but osteoporosis or a calcification shadow may be present; individuals had not received acupuncture in the preceding month; and a signed informed consent form.

### Exclusion criteria

The exclusion criteria are as follows: referred pain from the cervical spine, history of shoulder trauma, shoulder surgery, stoke, ipsilateral breast surgery, heart diseases and severe hypertension; osteoarthritis of the glenohumeral joint or systemic bone and joint disorder (rheumatoid arthritis); endocrine diseases such as hyperthyroidism; severe infection; and undergoing current therapy involving analgesics.

### Interventions

**Figure 1:**
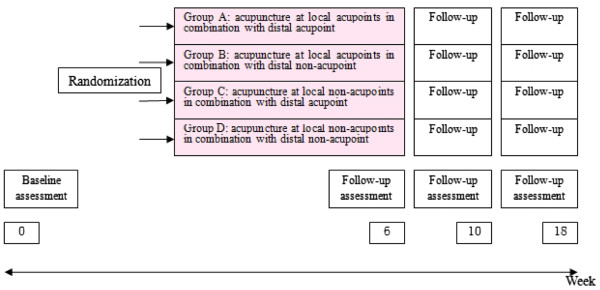
Flow chart.

The participating acupuncturists will be members who have been qualified for at least three years and who hold a Chinese medicine practitioner license from the Ministry of Health of the People’s Republic of China. The acupuncture treatments will be performed according to a treatment protocol already developed for this purpose. This will allow for each patient having a customized treatment within a standardized theory-driven framework. All patients will have a standardized interview and receive more information about the study and treatments. Patients are informed in a manner suggesting that four different types of acupuncture treatment are compared some are traditional and others are new. As the effect of all four is uncertain, terms such as ‘placebo’ or ‘sham’ were not used. Similar strategies of informed consent have been used in most previous acupuncture trials [7]. After obtaining informed consent from each participant and completing a baseline evaluation, they will receive 12 treatments of acupuncture, one to three times per week for six weeks in total. Time points are as shown in Figure 1.

Patients who meet the inclusion criteria are randomized to one of four treatment groups:

Group A will receive acupuncture at local acupoints in combination with distal acupoint.

Group B will receive acupuncture at local acupoints in combination with distal non-acupoint.

Group C will receive acupuncture at local non-acupoints in combination with distal acupoint.

Group D will receive acupuncture at local non-acupoints in combination with distal non-acupoint.

**Figure 2:**
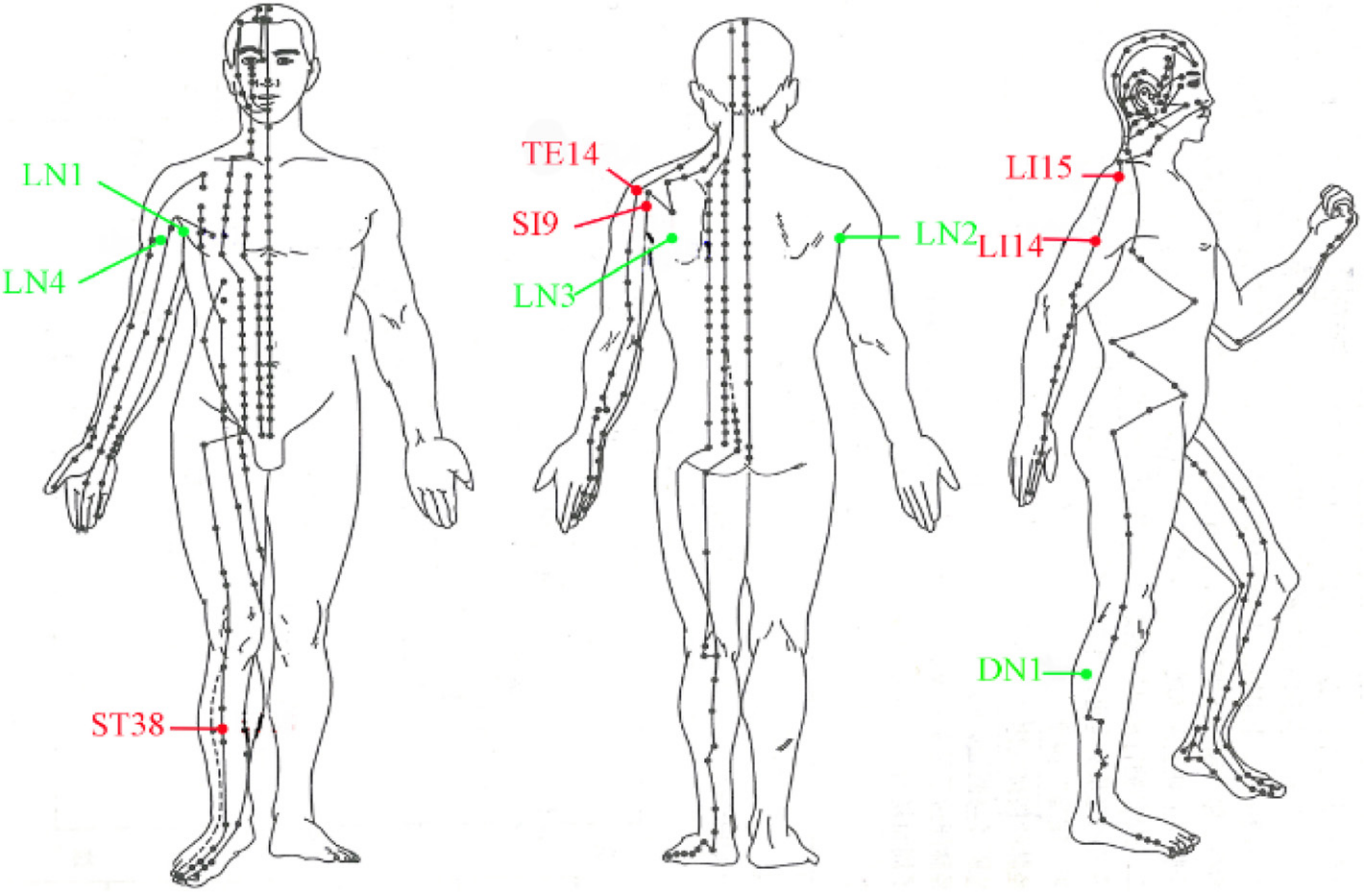
The points used in the trial.

We selected local acupoints and local non-acupoints on the same side of the shoulder lesion, while distal acupoint and distal non-acupoint were opposite to the shoulder lesion. Acupuncture points are selected as shown in Figure 2. Local acupoints: Jianyu (LI 15), Jianliao (TE 14), Jianzhen (SI 9), Binao (LI 14). Distal acupoint: Tiaokou (ST 38). Local non-acupoints (LN): (1) anterior axillary fold; (2) posterior axillary fold; (3) in the shoulder department, 2 cm below Tianzong (SI 11); (4) inside of the upper arm side, Tianfu (LU 3) inward 1 cm, between pericardium meridian and lung meridian. Distal non-acupoint (DN): lateral to the shank, 3 cun below Yanglingquan (GB34), the midway between gall bladder meridian and bladder meridian.

We will use sterile, prepacked needles (Huatuo disposable acupuncture needle, Suzhou Medical Co. Ltd., Jiangsu, China). The sizes to be used are 0.25 mm × 40 mm and 0.25 × 75 mm. The doctor will first insert a needle into the distal acupoint or distal non-acupoint (during the needling of these distal points a brief movement of the shoulder is allowed) for 20 minutes, and then insert a needle into the local acupoints or local non-acupoints for a further 20 minutes.

The doctor should ensure the patient is kept in the sitting position, and insert a 0.25 mm × 75 mm acupuncture needle from ST 38 to BL 57 (Chengshan). The needle will be rotated up and down to induce a needle sensation (called ‘de qi’) for 30 seconds. At the same time, the patient will be instructed to demonstrate a small amount of range of motion in the shoulder, such as abduction, external rotation and elevation. Local acupoints will be inserted 10 to 15 mm in depth with a 0.25 mm × 40 mm acupuncture needle. Local non-acupoints and distal non-acupoint will be inserted less than 5 mm in depth with a 0.25 mm × 40 mm acupuncture needle.

### Randomization and blinding

This is a block randomized single blind 2 × 2 factorial design. Randomization is to be conducted by a statistics specialist who will have no contact with the participants. Random numbers with block randomization are generated with SAS software (SAS Institute, Inc., Cary, NC, USA). Participants will be allocated to one of the four groups at a ratio of 1:1:1:1. The research coordinator, who will not be involved with data collection, will inform the acupuncturist of the treatment assignment via the telephone.

The patients, data collection staff, and data analysts will be blinded during the study period. Acupuncturists are not blinded to the treatments they deliver due to the nature of the intervention. During the intervention, the acupuncturist and personnel who collect data will be segregated immediately after the start of treatment and are instructed not to exchange information with each other.

### Primary outcome measures

Shoulder pain intensity will be graded using a 100-mm Visual Analogue Scale (VAS) by patients themselves, with 0 representing ‘no pain at all’ and 100 mm representing ‘the most intense pain imaginable’ [4]. The assessment is at baseline (before treatment initiation), 6 weeks after the first acupuncture, 10 weeks after the first acupuncture and 18 weeks after the first acupuncture.

### Secondary outcome measures

There will be three secondary outcome measures. First, functions of the shoulder joint will be evaluated using the Constant-Murley score (CMS). This consists of four domains: pain (one item), activities of daily living (ADL; three items for activity level, such as work, sports and sleep), one item for hand positioning (rotation), mobility (four items: forward and lateral abduction and elevation, external and internal rotation), and power or strength (one item). Pain and ADL 1 to 3 are interviewed from the patient (self-assessed); all other items are assessed by examiner [8]. The assessment will be performed at baseline (before treatment initiation), 6 weeks after the first acupuncture, 10 weeks after the first acupuncture, and 18 weeks after of the first acupuncture. Second, the quality of life is assessed using Short form-36 (SF-36) [9]. The assessment is at baseline (before treatment initiation), 6 weeks after the first acupuncture, 10 weeks after the first acupuncture and 18 weeks after the first acupuncture. Third, the perceived credibility of acupuncture is evaluated by The Treatment Credibility Scale (TCS) [10] after a 6-week acupuncture session. It is a five-item questionnaire ranging from 1 (not at all) to 5 (very confident). Items are averaged to provide a single treatment credibility score, with high scores reflecting high treatment credibility.

Participants will also be advised to report any adverse events they experience, including discomfort or bruising at the sites of needle insertion, nausea, or feeling faint after a 6-week acupuncture treatment.

### Sample Size

According to the previous pilot study [4],an average 20-point reduction on the VAS scores relating to shoulder pain has a clinically significant difference, and percentages of responders for the primary endpoint are verum 65%, sham 24%. The following formula is used for a four-group trial [11]:

$$n=\frac{2\lambda }{{\left(2\mathrm{si}n^{‒1}\sqrt{P_{\text{max}}}‒2\mathrm{si}n^{‒1}P_{\text{min}}\right)}^{2}}$$

Based on 0.9 power to detect a significant difference (α = 0.05,ν = 4-1 = 3,λ =14.17), the required sample size is 34 patients in each group. Allowing for 20% attrition, we should recruit 164 patients, with 41 in each group.

### Data analysis

Statisticians who are independent from the research team will analyze data in accordance with a 2 × 2 randomized factorial study design. Every analysis is to be conducted using the software of SPSS 12.0. The statistical model includes two fixed factors (local acupoints and distal acupoints). Interaction is to be evaluated by the interaction term in the analysis of variance (ANOVA) model and visual assessment of profile plots.

## Discussion

We have presented the design and protocol for the randomized controlled trial of acupuncture at local and distal points for CSP. Completion of this trial will help to identify whether acupuncture at local acupoints in combination with distal acupoints are more effective than local acupoints or distal acupoints alone.

A Cochrane systematic review of acupuncture for shoulder pain concluded that there is not enough published evidence of sufficient quality to support or refute its use [12]. A trial of acupuncture for shoulder pain concluded that acupuncture is an effective long-term treatment for patients with shoulder pain (from soft-tissue lesions) in a primary care setting [13]. Typically acupuncturists often use a combination of local and distal points to treat a condition such as CSP. According to experience from our clinical practice, we consider that distal points could improve range of motion while local points could be effective in providing pain relief. Therefore we hypothesize that acupuncture at local acupoints in combination with distal acupoints may be more effective than needling points separately to treat CSP. This project will not only increase the knowledge about the effects of acupuncture treatment for CSP, but also confirm the effect of a combination of local and distal acupoints.

## Trial status

This trial is currently recruiting participants.

## Abbreviations

CSP: Chronic shoulder pain; CMS: Constant-Murley score; SF-36: Short form-36; TCS: Treatment Credibility Scale; VAS: Visual Analogue Scale.

## Competing interests

The authors declare that they have no competing interests.

## Authors’ contributions

CZL: conception and design, critical revision for important intellectual content and final approval of the manuscript. QNF: conception and design, drafting the manuscript and final approval of the manuscript. GXS: conception and design, drafting the manuscript and final approval of the manuscript. QQL: data collection and analysis and final approval of the manuscript. TH: data collection and analysis and final approval of the manuscript. BZL: conception and design, final approval of the manuscript. SFS: data collection and analysis and final approval of the manuscript. JW: data collection and analysis, final approval of the manuscript. CT: data collection and analysis and final approval of the manuscript. BFY: data collection and analysis and final approval of the manuscript. All authors have read and approved the manuscript.
